# Supplementary material for: Self-management education for hypertension, diabetes, and dyslipidemia as major risk factors for cardiovascular disease: Insights from stakeholders’ experiences and expectations
Source: PLoS One. 2024 Sep 26;19(9):e0310961. doi: 10.1371/journal.pone.0310961 (PMC11426497; doi:10.1371/journal.pone.0310961)
Supplement: S1 Checklist — (DOCX) [file pone.0310961.s001.docx]

**COREQ Checklist(**Consolidated criteria for reporting qualitative research)

**Domain 1: Research team and reflexivity Personal Characteristics**

1. Interviewer/facilitator: Which author/s conducted the interview or focus group? NS conducted data collection, including interviews and FGDs, while MM supervised the FGDs.

2. Credentials: What were the researcher’s credentials? E.g. PhD, MD N

3. Occupation: What was their occupation at the time of the study?

4. Gender: Was the researcher male or female?

5. Experience and training: What experience or training did the researcher have?

2,3,4,5: This paper presents findings from the MD thesis of Nazanin Soleimani (Female). Masoud Mirzaei (Male), supervisor of the research team, holds MD, MPH, and PhD degrees in cardiovascular disease prevention and has completed a postdoctoral fellowship. Fatemeh Ebrahimi (Female) acted as the thesis co-supervisor and holds a PhD in Sociology. MM and FE have significant expertise in qualitative research, particularly in this field, and provided training and supervision to NS throughout the study. NS conducted interviews and FGDs under the supervision of both team members. Further details regarding contributions are provided in the author contribution section.

**Relationship with participants**

6. Relationship established: Was a relationship established prior to study commencement? NO

7. Participant knowledge of the Interviewer: What did the participants know about the researcher? e.g. personal goals, reasons for doing the research: They knew about research goals and signed an informed consent.

8. Interviewer characteristics: What characteristics were reported about the interviewer/facilitator? e.g. Bias, assumptions, reasons and interests in the research topic: The interviewer, Nazanin Soleimani, was introduced to participants as a physician-researcher conducting a study under the supervision of the Health Center. Patients were informed that their self-management would be evaluated during the interview, and necessary education and guidance would be provided to them.

**Domain 2: study design Theoretical framework**

9. Methodological orientation and Theory: What methodological orientation was stated to underpin the study? grounded theory

Participant selection

10. Sampling: How were participants selected? Purposive sampling

11. Method of approach: How were participants approached? Interviews were conducted in person or by telephone, depending on individual preference.

12. Sample size: How many participants were in the study? 19 patients with hypertension, type 2 diabetes mellitus, and dyslipidemia, 11 primary healthcare providers, and five provincial health policymakers and health managers

13. Non-participation: How many people refused to participate or dropped out? Reasons? none

Setting

14. Setting of data collection: Where was the data collected? Primary healthcare centers

15. Presence of non-participants: Was anyone else present besides the participants and researchers? NO

16. Description of sample: What are the important characteristics of the sample? Interviews were conducted with patients selected from six PHCs, chosen for their diverse cultural, economic, and social contexts and their high client load and accessibility. Characteristics were discussed in the results section.

17. Interview guide: Were questions, prompts, guides provided by the authors? Was it pilot tested?YES. Three pilot interviews were conducted and reviewed to revise the primary interview guide. Guidelines for conducting interviews and discussions were developed based on the literature review and professional consensus and were provided in supporting information files.

18. Repeat interviews: Were repeat interviews carried out? If yes, how many? NO

19. Audio/visual recording: Did the research use audio or visual recording to collect the data? Audio recorded

20. Field notes :Were field notes made during and/or after the interview or focus group? YES

21. Duration :What was the duration of the interviews or focus group? Interviews, averaging between 30 and 90 minutes, Two FGDs were conducted, one with primary health care providers and one with provincial HPMs and HMs, each lasting approximately 90 minutes

22. Data saturation: Was data saturation discussed? Interviewing and concurrent coding continued until theoretical saturation was reached.

23. Transcripts returned: Were transcripts returned to participants for comment and/or correction? NO

**Domain 3: analysis and findings**

Data analysis

24. Number of data coders: How many data coders coded the data? two

25. Description of the coding tree: Did authors provide a description of the coding tree? Yes

26. Derivation of themes :Were themes identified in advance or derived from the data? From the data

27. Software: What software, if applicable, was used to manage the data? ATLAS.ti 9

28. Participant checking: Did participants provide feedback on the findings? YES. Health professionals provided feedback.

Reporting

29. Quotations presented: Were participant quotations presented to illustrate the themes / findings? Was each quotation identified? YES

Data and findings:

30. consistent : Was there consistency between the data presented and the findings? YES

31. Clarity of major themes: Were major themes clearly presented in the findings? YES

32. Clarity of minor themes: Is there a description of diverse cases or discussion of minor themes? YES
