# Supplementary material for: Self-management education for hypertension, diabetes, and dyslipidemia as major risk factors for cardiovascular disease: Insights from stakeholders’ experiences and expectations
Source: PLoS One. 2024 Sep 26;19(9):e0310961. doi: 10.1371/journal.pone.0310961 (PMC11426497; doi:10.1371/journal.pone.0310961)
Supplement: S1 Table — (S1A Table: Semi-Structured Interview Guide for Patients, S1B Table: Focus group discussion Guide for Primary healthcare providers, S1C Table: Focus group discussion Guide for provincial health policymakers and health managers). (DOCX) [file pone.0310961.s002.docx]

| Interview Questions | Guiding and Prompting Questions |
| --- | --- |
| 1. Could you please describe the illness(es) you are experiencing and share your experience with managing and controlling them? | --- |
| 1. What do you think is the concept of chronic disease self-management or self-care, and what does it include? | - Have you ever heard about "chronic disease self-management"? If so, please explain what you know. If not, what comes to mind when you hear "chronic disease self-management"? - Which of the activities you undertake due to your illness do you think fall under the concept of "chronic disease self-management"? |
| 1. How and through what means did you learn to manage your illnesses and health condition? | - What methods and resources have you used to learn how to manage and control your illness? - Do you have specific skills for managing and controlling your illnesses? How did you learn these skills? - When you have questions about your illness or illnesses, what do you do? |
| 1. Do you have any experience with training or educational support for managing and controlling your illnesses? | - How do you evaluate the training and educational support you receive at healthcare centers regarding your illnesses and their management? - Have you received any training or educational support regarding your chronic disease(s) at primary healthcare centers? Please elaborate on your experiences, including any positives and negatives. |
| 1. What do you think the training and educational support for managing and controlling your disease should include, and how should it be provided? | - How and where do you think this training and educational support should be provided to you? - Who do you think should provide you this training and educational support? - What questions have you had or do you have about your disease(s) and their management? |
| 1. What factors do you think influence the effectiveness of training you receive about self-management, as well as your ability to learn and implement it? |  |
| 1. What is your opinion on non-face-to-face methods for learning self-management of chronic diseases, such as telephone-based approaches, internet and social media resources, or mobile applications? |  |

S1A Table: Semi-Structured Interview Guide for Patients

*(Note that this paper presents findings from a larger research project. Although all these questions were covered in the interviews and coded and analyzed, this paper specifically focuses on relevant sections, and data from some of these questions are not addressed here.)*

S1B Table: Focus group discussion Guide for Primary healthcare providers

*(Note that this paper presents findings from a larger research project. Although all these questions were covered in the focus group discussion and coded and analyzed, this paper specifically focuses on relevant sections, and data from some of these questions are not addressed here.)*

| **Question category** | **Question** | Guiding and Prompting Questions |
| --- | --- | --- |
| Opening Question | 1. In your opinion, what does the conceptual definition of educational support for chronic disease self-management include? |  |
| Transition Question | 1. What experiences have you had in primary healthcare centers regarding educational support for chronic disease self-management? |  |
| Key Question | 1. What knowledge and skills do you think patients with chronic diseases such as diabetes, hypertension, and dyslipidemia need for managing and controlling their conditions? 2. What factors do you think influence the educational support for chronic disease self-management provided in primary healthcare centers and its learning and implementation by patients? |  |
|  | 1. What barriers and challenges have you experienced regarding educational support for self-management in primary healthcare centers? 2. Do you have any solutions for the existing challenges? | - What obstacles related to facilities, planning, patients, policymakers, etc., have you encountered? |
|  | 1. What is your opinion on the different methods of educational support for chronic disease self-management? | - What is your opinion on non-face-to-face methods of educational support for chronic disease self-management, such as telephone-based support, internet resources, social media, or mobile applications? |
| Ending Question | 1. Considering the research objectives, is there any aspect that you believe has been overlooked and needs to be discussed? |  |

S1C Table: Focus group discussion Guide for provincial health policymakers and health managers

*(Note that this paper presents findings from a larger research project. Although all these questions were covered in the Focus group discussion and coded and analyzed, this paper specifically focuses on relevant sections, and data from some of these questions are not addressed here.)*

| **Question category** | **Question** | Guiding and Prompting Questions |
| --- | --- | --- |
| Opening Question | 1. In your opinion, what does the conceptual definition of educational support for chronic disease self-management include? |  |
| Transition Question | 1. What experiences do you have regarding the planning, policy-making, and implementation of educational support for chronic disease self-management in primary healthcare centers? |  |
| Key Question | 1. What knowledge and skills do you think patients with chronic diseases such as diabetes, hypertension, and dyslipidemia need for managing and controlling their conditions? 2. What factors do you think influence the educational support for chronic disease self-management provided in primary healthcare centers and its learning and implementation by patients? |  |
|  | 1. What is your opinion on the different methods of educational support for chronic disease self-management? | - What is your opinion on non-face-to-face methods of educational support for chronic disease self-management, such as telephone-based support, internet resources, social media, or mobile applications? |
|  | 1. What barriers and challenges have you encountered in the planning, policy-making, and implementation of educational support for self-management in primary healthcare centers? Alternatively, what barriers and challenges do you anticipate? 2. Do you have any solutions for the existing or anticipated challenges? |  |
| Ending Question | 1. Considering the research objectives, is there any aspect that you believe has been overlooked and needs to be discussed? |  |
