# Supplementary material for: Self-management education for hypertension, diabetes, and dyslipidemia as major risk factors for cardiovascular disease: Insights from stakeholders’ experiences and expectations
Source: PLoS One. 2024 Sep 26;19(9):e0310961. doi: 10.1371/journal.pone.0310961 (PMC11426497; doi:10.1371/journal.pone.0310961)
Supplement: Attachment — Submitted filename: response to reviewer.docx [file pone.0310961.s004.docx]

**Dear Editor,**

We sincerely appreciate the opportunity to submit the revised version of our manuscript titled "Self-management education for hypertension, diabetes, and dyslipidemia as major risk factors for cardiovascular disease: Insights from stakeholders' experiences and expectations" for review in *PLOS ONE*. We are grateful for the time and effort you and the reviewer have invested in providing valuable feedback on our paper.

We have carefully considered all the comments and suggestions the reviewer provided and made significant revisions to address them.

In the following sections, we provide a detailed response to the reviewer's comments and concerns. We are confident that our revisions adequately address the feedback provided, and we believe that the manuscript now meets the standards required for publication.

**Best regards,**

**Masoud Mirzaei**

**Response to reviewer comments:**

We want to express our gratitude for your time and effort in providing valuable feedback on our paper. We have thoroughly reviewed your comments and suggestions and made revisions to address them.

The following section presents a detailed response to comments and concerns. We have also used highlights to describe the changes made in the main manuscript in response to your feedback.

| Comments | Our Response |
| --- | --- |
| 1. The second sentence of the abstract results should clarify that it concerns the patients group. It also requires rephrasing since it claims that most completed primary school but gives the percentage of just 32%. | Thank you for your comments. We have edited and clarified the abstract results and included detailed characteristics of both patients and health professionals.  *"Most patients were women (68%) aged 50-60 years (37%), with education levels from illiterate to master's degree; 32% had completed primary school. Most were housewives (52%), and 12 had multiple chronic diseases. Healthcare providers included six community health workers and five primary care physicians, with average experience of 12 and 19 years, respectively. Health policymakers and managers averaged 25 years of experience."* |
| 2. The abstract results present the subthemes without first presenting the themes. | We appreciate your comment. Due to the large number of sub-themes identified in the interviews and focus group discussions, our focus was on presenting the main themes in the summary; therefore, the wording may have been unclear, giving the impression that the sub-themes were detailed. We have revised the summary to clearly highlight the main themes.  *"Patient interviews and FGDs resulted in 12 and 13 subthemes, respectively, with five subthemes common to both sources. These subthemes were grouped into broader main themes, including effective content design,' 'effective presentation and delivery,' 'characteristics and conditions of involved parties,' and 'educational needs,' collectively reflect the central concept of 'effective self-management education."* |
| 3. The abstract conclusion should be improved. Currently it has two general statements that were known to be true without this new study. Instead, summarize the findings, particularly that which is new | Thank you for your comment. We have revised the abstract conclusion. One explanation may be that due to the complexity and richness of our findings, we focused on summarizing the central findings in the abstract, which may not fully highlight the novel aspects of our study.  We believe the most important finding is that while the core concept of "effective self-management education" was consistent across stakeholder groups, our study revealed significant variation in the subthemes. This underscores the importance of considering diverse viewpoints and highlights that while overarching concepts may appear consistent, exploring the details of stakeholder perspectives is essential to understanding their nuanced opinions.  *"Although the core concept and its main themes were evident and consistent across stakeholder groups, significant variations in subthemes from each stakeholder emerged. This underscores the importance of considering diverse viewpoints and highlights that, while overarching concepts may seem uniform, exploring the details of stakeholder perspectives is crucial for understanding their nuanced opinions. Effective education should integrate these insights, focusing on tailored communication, interactivity, and active monitoring"* |
| Additional changes | We shortened the background and methods sections of the abstract to meet the word count limitations. |
| 4. The manuscript refers to supplementary materials, but I could not find them on the PONE website. | Thank you for your comment,We added Supplementary 1, 2, and 3, which include the translations of the Semi-Structured Interview Guide for Patients, the Focus Group Discussion Guide for Primary Healthcare Providers, and the Focus Group Discussion Guide for Provincial Health Policymakers and Managers. Please note that this paper presents findings from a larger research project. While all these questions were covered in the interviews and FGDs and coded and analyzed, this paper focuses explicitly on relevant sections, and data from some of these questions are not addressed in this paper. |
| 5. Introduction – the second sentence refers to deaths per year without stating the country or countries. | Thank you for your comment, We added a sentence and global statistics from a new reference (WHO) for accuracy and deleted the previous sentence.  *"Cardiovascular disease (CVD) is the predominant chronic disease and the leading cause of premature death in many societies (4-6). An estimated 17.9 million people died from cardiovascular diseases in 2019, representing 32% of all global deaths(7)*." |
| 6. The introduction provides no review of prior literature on this topic, no summary of missing information, no mention of specific goals of the study. | Thank you for your comment, We have edited the introduction to address this issue by including a review of prior literature, summarizing the gaps in existing knowledge, and clearly outlining the specific goals of the study.  *"A qualitative study by Lotte Timmermans et al. in Flanders, Belgium, involved semi-structured interviews with 16 patient-caregiver dyads using purposive sampling and inductive content analysis. The study developed the SILCQ model—Supporting, Involving, Listening, Coordinating, and Questioning—as a conceptual framework for primary care, emphasizing its use in creating effective roadmaps and toolkits to enhance self-management support for chronic patients(23).*  *In another study, Marta Gorina et al. conducted a qualitative study using focus groups to identify critical factors influencing the adoption of self-care behaviors in patients with coexisting diabetes, hypertension, and hypercholesterolemia. The study involved patients, nursing professionals, and family doctors from primary care centers. Content analysis revealed that the adoption of self-management recommendations is influenced by factors related to the patients, healthcare professionals, and the healthcare system. The findings suggest that healthcare professionals should consider these broader determinants when recommending self-care (24).*  *Dena Schulman-Green et al. conducted a qualitative meta-synthesis of 53 studies to identify factors affecting self-management in adults with chronic illness, focusing on diabetes and cardiovascular disease. Analyzing data from diverse participants (mean age 57) across 20 countries, the study identified five key categories: Personal/Lifestyle Characteristics, Health Status, Resources, Environmental Characteristics, and Health Care System. The findings suggest that understanding these factors can improve self-management assessments and inform tailored interventions to enhance health outcomes(25).*  *Although valuable research has been conducted in this area, gaps still need to be addressed. A qualitative systematic review by Bruno Rushforth et al. (2016) highlights the need for strategies beyond a 'one size fits all' model for managing type 2 diabetes, emphasizing that effective improvement strategies must address organizational and individual levels(26). Additionally, while studies underscore the importance of patient-centered interventions and consider patient preferences for education (14, 27, 28), qualitative research often explores these indirectly by identifying facilitators and barriers to self-management support (11, 22, 26, 29-33).. This research project aims to fill a gap in directly addressing patient needs and preferences through comprehensive approaches that incorporate diverse stakeholders' experiences, perceptions, and expectations in self-management education programs for hypertension, diabetes mellitus, and dyslipidemia."* |
| 7. The Methods do not describe a conceptual model for understanding the role of education in health behavior change or disease self-management. | Thank you for your comment. We added our conceptual model and explained it.  *"While grounded theory typically follows an inductive approach, we used the Corbin and Strauss model for self-management as a conceptual framework(35). This model, which organizes self-management into medical, behavioral, and emotional aspects, guided our data collection and analysis. This approach helped organize and contextualize our findings within a broader understanding of self-management, ensuring that our research contributes meaningfully to the field while remaining grounded in the data."* |
| 8. The Methods does not provide information on the development and specific questions in the patient interview. Such information is also lacking for the questions used in the focus groups. Perhaps some of this was in the Supplemental files that I cannot find? | Thank you for your comment, We have added supplementary 1, 2, and 3, which include translations of the semi structured interview guide for patients, the focus group discussion guide for primary care providers, and the focus group discussion guide for provincial health policymakers and managers. It is important to note that this paper presents the findings of a larger research project. While all of these questions were covered, coded, and analyzed in the interviews and FGDs, this paper explicitly focuses on relevant sections, and data from some of these questions are not addressed in this paper |
| 9. The data are said to be available but no information about accessing them is provided. | We added raw data as a supplementary file. The interviews and FGDs were conducted in Farsi, and the coding process was performed in the same language. Subthemes and themes were translated into English for this paper. As a result, the raw data is only available in Farsi ATLAS .ti files. |
| 10. The authors indicate that their sample sizes allowed them to reach saturation. This may be true of the 19 patients, but I am not so sure since the patients had different CV disorders. | Thank you for your comment. While our sample included patients with hypertension, dyslipidemia, and diabetes, we believe these conditions share significant similarities in terms of management. Additionally, many participants had more than one of these conditions, further supporting the interconnected nature of their experiences. As a result, we achieved theoretical saturation despite the diversity in specific diagnoses. |
| 11. I doubt the authors reach saturation with the primary providers since it included just 6 community health workers and 5 primary care providers. These two types of professionals typically have very different education and roles. The 5 health administrators are probably too few to report. | We appreciate your comment, We acknowledge that our study had limitations regarding the number of participants in the focus group discussions (FGDs), as detailed in the strengths and limitations section of our paper. Despite this, we believe the findings are valuable and warrant inclusion. We considered these FGDs as a unified component of the study aimed at exploring the experiences and expectations of healthcare professionals, rather than as separate parts. Therefore, we coded them together.  We explained this in a paragraph on our paper:  *"We treated these FGDs as a unified component of the study focused on exploring the experiences and expectations of health workers, rather than as separate parts. As a result, we coded them together. Specifically, in Iran, primary health care centers consist of primary care physicians and community health workers (CHWs), known as "Behvarz" in Farsi, who work as an integrated team. CHWs are full-time employees within the health system and are considered health care providers. They provide primary health care and work closely with community members to address the social determinants of health. Because of this collaborative approach, the roles of primary care physicians and CHWs overlap significantly, making their combined input critical to understanding primary health care."* |
| 12. Related to the above, in the US CHWs are not considered to be "health care providers." Please explain their respective roles in Iran and justify combining them in a single focus group. | Specifically, in Iran, primary health care centers consist of primary care physicians and community health workers (CHWs), known as "behvarz" in Farsi, who work as an integrated team. CHWs are full-time employees within the health system and are considered health care providers. They provide primary health care and work closely with community members to address the social determinants of health. Because of this collaborative approach, the roles of primary care physicians and CHWs overlap significantly, making their combined input critical to understanding primary health care. |
| 13. On lines 143-145, the authors mention use of "specific research objectives" but these are not listed or explained. | We replaced "specific research objects" with "to capture the distinct experiences and expectations of patients and health professionals as two different stakeholders". |
| 14. Methods -- Were all the interviews in Persian/Farsi? Describe how translation to English was done and whether this occurred. before or after coding? | Thank you for your comment, We have added the following text under the Data Collection subheading of the Methods section:  *"All interviews and FGDs were conducted in Persian (Farsi), and the coding process was also carried out in Farsi. After completing the coding, the sub-themes and themes were translated into English by the research team, including one researcher who is a native speaker of both Farsi and English. This translation process was followed by a validation session where the translated terms were discussed and verified using relevant literature and dictionaries to ensure accuracy and consistency."* |
| 15. In qualitative research, coding is done by two individuals. This study used just one, which appears to be a weakness. Please address. | We value your comment but The coding was not done by just one researcher. In this study, coding was conducted by two separate researchers.We have mentioned this point under the subheading Coding Process in the Methods section.  *"Two researchers did coding, with initial codes refined in online sessions between them. This was followed by a team session to review and finalize the codes and themes, ensuring a thorough and validated process."* |
| 16. Results – Table 2 is redundant with Table 1 and can be removed. | We appreciate your comment but Table 2 is not redundant with Table 1. Table 1 summarizes the cumulative characteristics of patients, while Table 2 presents the frequency of specific patient characteristics. This distinction is critical because Table 2 highlights individual characteristics that are important for understanding the nuances of each patient's profile. Including both tables provides a comprehensive view of the data, enriching the analysis by detailing individual patient characteristics in addition to cumulative statistics. |
| 17. The figure is creative, and I like it. However, is it true that the bottom right is blank (no patient options on "Effective presentation and delivery")? | The figure illustrates the specific subthemes derived from both patient and healthcare professional groups, as well as the overlap of subthemes across themes. The "Effective Presentation and Delivery" section has no unique subthemes for patients. This section contains only the common subthemes-"Teaching Methodology and Materials" and "Accessibility"-that overlap with those identified by health professionals. The blank appearance in the lower right for patient-specific subthemes does not indicate that no subthemes were derived from patient interviews about effective presentation and delivery; rather, it reflects that these subthemes are shared with healthcare professionals. |
| 18. The Discussion paragraph on limitations should also mention limited generalizability outside of Iran and countries with similar cultures and medical systems. | Thank you for your comment:Regarding your comment, we have made the following changes to the paper:  "Given the need for improved chronic disease SM education and the current lack of evidence, the findings are relevant to Iran and similar low- and middle-income contexts. However, their generalizability is limited due to the qualitative nature of the research." |
| Additional changes | We replaced 'educators' with 'instructors' throughout the text to ensure consistency. |
